# Preventing postpartum insomnia: findings from a three-arm randomized-controlled trial of cognitive behavioral therapy for insomnia, a responsive bassinet, and sleep hygiene

**DOI:** 10.1093/sleep/zsae106

**Published:** 2024-05-13

**Authors:** Nina Quin, Liat Tikotzky, Laura Astbury, Marie-Antoinette Spina, Jane Fisher, Lesley Stafford, Joshua F Wiley, Bei Bei

**Affiliations:** Turner Institute for Brain and Mental Health, School of Psychological Sciences, Faculty of Medicine, Nursing and Health Sciences, Monash University, Melbourne, VIC, Australia; Women’s Mental Health Service, Royal Women’s Hospital, Melbourne, VIC, Australia; Department of Psychology, Ben-Gurion University of the Negev, Be’er Sheva, Israel; Turner Institute for Brain and Mental Health, School of Psychological Sciences, Faculty of Medicine, Nursing and Health Sciences, Monash University, Melbourne, VIC, Australia; Turner Institute for Brain and Mental Health, School of Psychological Sciences, Faculty of Medicine, Nursing and Health Sciences, Monash University, Melbourne, VIC, Australia; Global and Women’s Health, School of Public Health and Preventive Medicine, Monash University, VIC, Australia; Women’s Mental Health Service, Royal Women’s Hospital, Melbourne, VIC, Australia; Melbourne School of Psychological Sciences, University of Melbourne, VIC, Australia; Turner Institute for Brain and Mental Health, School of Psychological Sciences, Faculty of Medicine, Nursing and Health Sciences, Monash University, Melbourne, VIC, Australia; Turner Institute for Brain and Mental Health, School of Psychological Sciences, Faculty of Medicine, Nursing and Health Sciences, Monash University, Melbourne, VIC, Australia; Women’s Mental Health Service, Royal Women’s Hospital, Melbourne, VIC, Australia

**Keywords:** insomnia, pregnancy, cognitive behavioral therapy, pediatrics—infants, women’s health, postpartum depression

## Abstract

**Study Objectives:**

Insomnia symptoms are common during the perinatal period and are linked to adverse outcomes. This single-blind three-arm randomized-controlled trial examined whether two interventions targeting different mechanisms prevent postpartum insomnia.

**Methods:**

Participants were nulliparous females 26–32 weeks gestation with Insomnia Severity Index (ISI) scores ≥ 8, recruited in Australia and randomized 1:1:1 to: (1) a responsive bassinet (RB) designed to support infant sleep and reduce maternal sleep disruption until 6 months postpartum, (2) therapist-assisted cognitive behavioral therapy for insomnia (CBT-I) delivered during pregnancy and postpartum, or (3) a sleep hygiene booklet (control; CTRL). Outcomes were assessed at baseline (T1), 35–36 weeks gestation (T2), and 2, 6, and 12 months postpartum (T3–T5). The primary outcome was ISI scores averaged T3–T5. Primary analyses were regressions controlling for baseline outcomes.

**Results:**

One hundred and twenty-seven participants (age M ± SD = 32.62 ± 3.49) were randomized (RB = 44, CBT-I = 42, CTRL = 41). Both interventions were feasible and well-accepted, with few related adverse events reported. Compared to CTRL, the average ISI across T3–T5 was lower for CBT-I (*p* = .014, effect size [ES] = 0.56, medium) but not RB (*p* = .270, ES = 0.25, small). Exploratory findings on maternal insomnia diagnosis, sleep disturbance, sleep-related impairment, beliefs and attitudes about sleep, depression, anxiety, as well as infant sleep outcomes were also presented.

**Conclusions:**

CBT-I but not RB reduced prenatal insomnia (very large effect) and prevented postpartum insomnia (medium effect). Further research is needed to examine the effects of both CBT-I and RB on other outcomes such as sleep-related well-being, postpartum depression, and maternal postpartum sleep duration.

**Clinical Trial Registration:**

The Study for Mother-Infant Sleep (The SMILE Project): reducing postpartum insomnia using an infant sleep intervention and a maternal sleep intervention in first-time mothers. https://www.anzctr.org.au/Trial/Registration/TrialReview.aspx?id=377927, Australian New Zealand Clinical Trials Registry: ACTRN12619001166167.

## Graphical Abstract

**Graphical Abstract:**
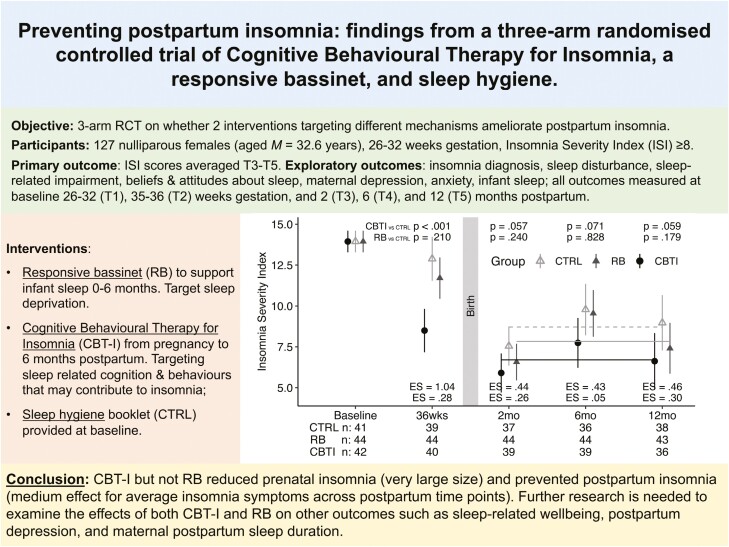

**Statement of Significance**

This randomized-controlled trial investigated whether two interventions targeting different mechanisms (i.e. infant-related maternal sleep disruption as a precipitator, and unhelpful sleep-related cognitions/behaviors as perpetuators) ameliorate postpartum insomnia. Compared to a control condition, CBT-I (but not the responsive bassinet condition) prevented postpartum insomnia, with medium-sized effects.

## Insomnia in the Perinatal Context

Insomnia symptoms include difficulties initiating and maintaining sleep, and/or early morning awakenings despite adequate sleep opportunities [1]. Approximately 23% of the adult population report insomnia symptoms, and for 10%–15% of adults who meet diagnostic criteria for insomnia disorder [2], these symptoms are significant, persistent, and impair daytime functioning [3].

Approximately 50%–73% of birthing parents report changes to their sleep–wake patterns during the perinatal periods, and rates of self-reported insomnia symptoms range from 17% to 30% during pregnancy and postpartum [4–8]. Furthermore, half of birthing parents with clinically significant insomnia symptoms during pregnancy have persistent symptoms at 2 years postpartum [8].

Sleep disturbances and insomnia in birthing parents have been associated with pre-term birth [9], gestational diabetes [10], family dysfunction [11], fatigue [12], increased risk/symptoms of perinatal depression and anxiety [13, 14], and poorer mother-infant relationships [15]. Efforts to understand and address perinatal insomnia symptoms have been a priority in recent research. A widely accepted theory that explains how normal sleep can deteriorate into chronic insomnia which is also relevant for perinatal insomnia, is the Spielman model [16], otherwise known as the 3P behavioral model of insomnia [17].

## The 3P model of insomnia

The 3P model describes three components in the development of insomnia: predisposing, precipitating, and perpetuating factors. Predisposing factors increase vulnerability for insomnia (e.g. genetic predispositions) [18]. Precipitating factors trigger an acute period of sleep disruption, for example, stressful life events or health/physical changes [17]. While individuals attempt to cope with acute sleep loss, they may develop unhelpful thoughts (e.g. worries about sleep) and behaviors (e.g. increasing time in bed in an attempt to increase sleep) [19], which perpetuate sleep complaints after the precipitant has reduced.

Precipitating factors during the perinatal period include physical changes in pregnancy (e.g. fetal growth, hormonal changes, urination, and nausea), and these are often replaced by the numerous demands of overnight infant care postpartum [20–25]. As parents cope with these significant sleep disruptions, they can develop unhelpful sleep-related thoughts (e.g. “I will feel terrible tomorrow if I don’t go back to sleep soon”) and behaviors such as napping in the late afternoon–evening. When overnight infant care decreases, parents may continue to experience sleep difficulties due to unhelpful sleep-related thoughts and behaviors, contributing to chronic insomnia. The 3P model has allowed researchers to direct treatments toward modifiable precipitating and perpetuating factors, specifically (1) infant sleep as a major source of perinatal sleep disruption and (2) unhelpful sleep-related cognitions and behaviors as perpetuating factors are potential therapeutic targets for preventing and reducing insomnia in perinatal populations.

## The efficacy of maternal sleep interventions

Cognitive behavioral therapy for insomnia (CBT-I) is considered the first line treatment for individuals with insomnia symptoms and addresses unhelpful sleep-related cognitions and behaviors [26, 27]. Recent randomized-controlled trials have demonstrated that CBT-I administered in pregnancy increases insomnia disorder remission rates [28] and reduces insomnia symptoms at post-intervention and postpartum follow-ups among birthing parents [29–31]; a recent study showed that for birthing parents with insomnia symptoms during pregnancy, CBT-I administered in pregnancy and early postpartum can lead to lower insomnia symptoms, sleep-related impairment, and dysfunctional sleep beliefs and attitudes during the first two years postpartum [27]. Although CBT-I is an efficacious treatment for insomnia by targeting sleep-related cognitions/behaviors as perpetuating factors, CBT-I does *not* address infant sleep as a primary precipitant of perinatal sleep disturbance.

## The efficacy of infant sleep interventions

Infant sleep studies rarely document behavioral intervention effects for birthing parents’ sleep, and of those that did, improvements in maternal sleep were modest [32–36]. Meta-analyses and systematic reviews suggest that behavioral sleep interventions improve sleep onset duration and night awakening frequency/duration in infants with a mean age of 17.6 months [37], but do not reduce infant nighttime awakenings in infants younger than 6 months old [38, 39]. Infant sleep studies typically focus on infants aged >6 months old with mild to severe infant sleep problems [32–34] as infant sleep is not expected to consolidate until an infant is >6 months old [40]. However, it is common for parents to report distress regarding their infant’s sleep between 3 and 6 months postpartum [41], highlighting a need for sleep-specific support in the early postpartum period.

In the context of insomnia, limited infant sleep support in the first 6 months postpartum has hindered the effort to understand how perinatal insomnia symptoms could be mitigated by reducing exposure to a major precipitant from the birth of the infant (i.e. decreasing infant nocturnal wakefulness and reducing maternal sleep disruption overnight). “Responsive bassinets” (i.e. a bassinet that automatically responds to the infant’s cries) are designed for infants 0–6 months old, and may support early postpartum sleep and reduce infant-related sleep disruption from the birth of the infant. However, further research is needed regarding the efficacy of responsive bassinets, and it is unclear the extent to which perinatal insomnia symptoms could be mitigated by reducing infant-related sleep disruption as a major precipitating factor from the birth of the infant. Moreover, it is unclear whether two distinctive interventions targeting different potential mechanisms (i.e. infant sleep as a precipitator, and unhelpful sleep-related cognitions/behaviors as perpetuators) may ameliorate the development of postpartum insomnia.

## Current study

In first-time birthing parents (i.e. nulliparas) at risk of postpartum insomnia (i.e., having sub-clinical insomnia symptoms in pregnancy), this three-arm, efficacy RCT based on the 3P model aimed to (1) examine whether reducing exposure to infant-related sleep disruption, a major precipitator of postpartum insomnia, lead to lower postpartum insomnia symptoms; and (2) evaluate the efficacy of two interventions, one addressing infant-related maternal sleep disruption as a precipitator, the other targeting maternal sleep-related cognitions/behaviors as perpetuators, in ameliorating symptoms of insomnia (primary outcome) while exploring changes in other outcomes previously linked to perinatal insomnia symptoms (i.e. sleep quality, sleep-related impairment, and symptoms of depression and anxiety; secondary outcomes). Exploratory analyses evaluated intervention effects for the following conditions:

A. Responsive Bassinet (RB), an infant sleep intervention. This study used a “responsive bassinet” from 0 to 6 months postpartum to reduce maternal exposure to infant-related night awakenings and perinatal sleep disruption from the birth of the infant, a major precipitating factor of postpartum insomnia.
B. CBT-I, a maternal sleep intervention addressing unhelpful thoughts, beliefs, and behaviors around sleep, delivered from pregnancy to 6 months postpartum to address perpetuating factors of postpartum insomnia.
C. Sleep Hygiene control (CTRL), providing sleep hygiene and information without the active components of CBT-I.

To achieve the aims, group differences in primary and other outcomes were compared between RB and CTRL, as well as CBT-I and CTRL, with the hypothesis that both RB and CBT-I will be associated with lower postpartum insomnia symptoms. This study was not powered for a non-inferiority comparison between the CBT-I and RB conditions.

## Materials and Methods

This study was a three-arm, parallel-group, single-blind, superiority RCT. Methodologies are summarized below, with the full protocol published elsewhere [42]. The trial was prospectively registered with Australian New Zealand Clinical Trial Registry (ACTRN12619001166167). Ethical approvals were obtained from the Royal Women’s Hospital Human Research Ethics Committee. Reporting follows CONSORT 2010 guidelines, including CONSORT-SPI and—PRO, and CONSORT Extensions and Harms (see Supplementary Tables S3 and S4).

### Participants

Inclusion criteria were: (1) nulliparous individuals in the third trimester of pregnancy (i.e., 26–32 weeks gestation and no older children); (2) singleton pregnancy; (3) age ≥ 18 years; (4) able to read and write in English; (5) had regular access to a smartphone, email, and internet; and (6) Score > 7 on the Insomnia Severity Index (ISI) [43]. These criteria were chosen as prenatal insomnia predisposes individuals to postpartum insomnia [44], and early intervention for perinatal individuals with insomnia symptoms (i.e. ISI score > 7) [43] was shown to have sustained benefits for 2 years postpartum [27]. Nulliparas (not multiparas) were selected to reduce confounders such as sleep disruption related to caring for older child(ren).

Exclusion criteria were: (1) undertaking shift work during pregnancy or study participation; (2) significant symptoms of the following sleep disorders based on the Duke Structured Clinical Interview for Sleep Disorders (DSISD) [45]: narcolepsy, sleep apnea, periodic limb movement disorder, restless legs syndrome, circadian rhythm sleep disorders; (3) severe current psychopathology, including posttraumatic stress disorder, panic disorder (only if > 4 nocturnal panic attacks in the past month), substance abuse/dependence disorders; OR lifetime bipolar or psychotic disorders; OR having high risk of harm to self or others; (4) use medications or substances that directly affect sleep; (5) medical conditions that directly affect sleep.

### Randomization and blinding

Eligible participants were randomized 1:1:1 using a complete randomization sequence generated in advance. Block designs with varying block sizes of 3 and 6 were used. Random seeds were generated to assure allocation concealment and pre-guessing of the allocation sequence at the end of each block. Randomization was stratified by baseline ISI (< 15 and > 14). The randomization sequence was set up in REDCap by a member of the research staff who (1) was not involved in recruitment or delivery of intervention and (2) was not one of the study’s Principal Investigators. Follow-up measures were either self-completed or conducted by research staff who were blinded to the condition, which was achieved by limiting access to group-specific data on REDCap.

### Procedures

Pregnant individuals enrolled in Childbirth Education at a public hospital in Victoria, Australia, were invited via email to participate in “a research project that evaluates different approaches to improving sleep for first-time mothers who currently experience sleeping difficulties.” The email invitation contained a link to an online Participant Information and Consent Form, and interested participants provided informed consent via a web-based survey form. The study was also advertised in the general community in Australia (e.g., invitations to participate with an online link to the Participant Information and Consent Form were posted on relevant online forums and social media).

After providing informed consent, participants completed online screening questions regarding initial inclusion criteria (i.e., adult first-time parent with singleton pregnancy and a score >7 on the ISI) and the baseline questionnaires, followed by a telephone screening interview to determine eligibility. The interview contained the DSISD and other questions related to mental/physical health to assess exclusion criteria. Eligible participants were then randomized by the researcher who conducted the screening interview.

Online questionnaires were administered via REDCap at 5 time points: 26–32 weeks of gestation (T1), 35–36 weeks of gestation (T2), and 2 months (T3), 6 months (T4), and 12 months (T5) postpartum. Table 1 contains a timeline of assessments and intervention material delivery. All participants received a phone call from a researcher at T1 for orientation (varied in duration and content depending on condition), and at T3 for encouraging adherence and troubleshooting (~15 minutes). A final telephone call by a researcher blinded to group allocation was made to all participants to administer the DSISD Insomnia module at T4 (about 10 minutes).

**Table 1.** Timing of Assessments and Intervention.

	Pregnancy		Postpartum				
	T1 26–32 w (baseline)	T2 35–36 w	2 w*	T3 2 m	3 m*	T4 6 m	T5 12 m
Self-report questionnaires	X	X		X		X	X
Telephone (intervention)	X^a^			X^b^			
Telephone (assessment)	X					X	
*Intervention groups*							
A: Responsive bassinet			X	X	X	X	
B: CBT-I	X	X	X	X	X	X	
C: Control	X						

*Intervention only, no assessment. Assessment at T1 was conducted before intervention commenced. All participants undertook online questionnaires at T1–T5, and received a telephone call from a researcher at T1, T3, and T4.

^a^Telephone call varied in content and duration depending on intervention condition.

^b^Telephone call of 5–20 minutes for encouraging intervention adherence and troubleshooting. The responsive bassinet condition used the bassinet from the birth of the infant until ~6 months postpartum. CBT-I intervention materials were delivered 1 week before completing questionnaires. The control condition received materials after completing the baseline assessment at T1.

Following completion of assessments at T4 and T5, participants were sent a $30 AUD gift voucher as a token of appreciation ($60 AUD total in vouchers).

No formal stakeholder advisory committee was appointed for this study; recruitment process and the development of the CBT-I intervention were based on those used in a previous trial [27] with feedback from individuals with lived experience.

### Intervention conditions

All participants continued their usual perinatal care. Interventions were manualized and telephone scripts were developed by the research team to ensure consistency. All intervention calls were recorded, and an independent rater (MAS) assessed intervention fidelity on 10% of randomly selected participants. Intervention fidelity was high for both CBT-I (99.5%) and RB (100%) groups. Participants in the RB condition and control condition were offered the CBT-I materials following the completion of the final assessment.

#### Infant Sleep Intervention.

The infant sleep condition received a “responsive bassinet” (RB) to use from the birth of the infant until 6 months postpartum (see Protocol for details of intervention condition) [42]. The RB is designed to calm crying and consolidate infant sleep by automatically responding to the infant’s cries. The bassinet is based on the soothing techniques called the “5S’s” (swaddling, side stomach, shushing white noise, sucking, and swinging) developed by Karp [46], which has been shown to significantly reduce the frequency of infant nighttime awakenings, increase daily sleep duration, facilitate infant soothing, and enhance parental self-efficacy [47–50]. The bassinet employs the “5S’s” by (1) automatically emitting three specially engineered white-noise sounds, (2) safely swaddling the infant in a secure swaddle that prevents rolling to an unsafe position during sleep, and (3) providing rhythmic “rocking” motions when crying noises are detected. Importantly, the bassinet employs the “5S’s” for a maximum of 3 minutes to provide the infant with the opportunity to transition between sleep cycles. If the infant does not settle within this time, the bassinet alerts adults for additional assistance (e.g. feeding and changing). There were no anticipated risks of harm for participants using the responsive bassinet. This bassinet is available for retail purchase, has been approved for Regulatory Compliance Mark in Australia, meets all American Academy of Pediatrics Safe Sleep recommendations, and has been approved by the Food and Drug Administration (FDA) Consumer Product Safety Commission as a medical device for infant sleep after the FDA completed data reviews [51].

Participants who received the bassinet were asked to use a mobile application linked with the bassinet, which (1) provided daily sleep reports on the infant’s sleep progress, (2) alerted parents that the infant requires additional soothing if the infant does not settle after 3 minutes of crying, (3) allowed parents to customize the motion and white noise settings for their infant’s individual needs, and (4) contained specialized settings to assist parents in transitioning their infant to a standard crib around 6 months of age. At approximately 5 months postpartum, participants were emailed information regarding how to transition their infant to a standard crib, a process that typically occurs over 1–2 weeks according to the bassinet manufacturer. Participants were not prescribed a specific time to transition their infant out of the bassinet, and rather, were encouraged to consider transitioning when their infant was 5–6 months old, or when their infant could get up on their hands/knees, whichever occurred sooner. To ensure the bassinet can accommodate larger 6-month-old infants, the bassinet was designed to fit an infant up to 33 pounds, with a length equal to that of a 9-month-old infant in the 98th percentile. If participants required additional information or support for the bassinet, the manufacturer’s customer care staff provided telephone consultations as with any commercial purchase so findings could approximate real-world use.

#### Maternal Sleep Intervention (CBT-I).

The maternal sleep condition used therapist-assisted self-help CBT-I to address unhelpful sleep-related cognitions and behaviors. The following evidence-based therapeutic components were included: psychoeducation about sleep processes, education on infant and parent sleep patterns, stimulus control, healthy sleep attitudes and behaviors, sleep hygiene, relaxation, skills for managing pain and discomfort, differentiating insomnia and sleep deprivation, managing unhelpful thoughts and worries, fatigue management, prioritizing self-care, and enlisting social support. Time-in-bed (TIB) restriction was not included in written materials, but administered over the phone if necessary. The necessity of time-in-bed (TIB) restriction was assessed during telephone contact with a researcher at T1 and T3, who calculated sleep efficiency (percentage of average TST against TIB based on self-report over the past week). If an individual presented with insomnia symptoms and a sleep efficiency < 80%, TIB restriction was utilized with the following steps: (1) consistent with previous recommendations [28], TIB recommendations were modified for pregnancy and postpartum and based on TST plus 30 minutes for pregnancy, and TST plus 30 minutes plus time spent caring for the infant at night during postpartum. For example, if a participant’s TST in pregnancy was 6.5 hours, then the recommended TIB was 7 hours; (2) TIB recommendations were always greater than or equal to 5.5 hours; (3) TIB recommendations were made in collaboration with participants. For example, participants chose the position of their sleep window based on their daily schedule/preferences (e.g. choosing to stay up later in the evening and wake up later in the morning); (4) participants were encouraged to avoid napping unless presented with significant daytime sleepiness or concerned about safety while driving; (5) TIB restriction was briefly reviewed with the participant 1–2 weeks after TIB recommendations commenced. Adjustments to TIB recommendations were made based on current sleep efficiency. If sleep efficiency was <80%, a new TIB recommendation was calculated and discussed, and if sleep efficiency was ≥80%, TIB recommendations were either not modified or extended by ~15 minutes, depending on the participant’s preferences/current daytime sleepiness. Participants were offered subsequent check-ins if required, and/or encouraged to manage their sleep routine independently moving forward if sleep efficiency was ≥80%.

Content of the intervention was delivered via: (1) A 50-minute individual telephone session conducted by one provisional psychologist (NQ) at T1 to introduce core intervention components and tailor strategies based on the individuals; up to three brief follow-up calls of ~15 minutes were made to those who required TIB restriction. A follow-up call at T3 (~15 minutes duration) was conducted to encourage adherence and troubleshooting; (2) A series of emails containing intervention components were delivered at T1–T4 (same time points as T1–T4 assessments), and in addition at 2 weeks and 3 months postpartum (Table 1). In total, participants received 20 intervention emails.

#### Control Condition.

This condition accounted for the nonspecific effects of participating in a sleep-related research project (e.g. contact with health professionals, receiving health information, and expectations of benefit). Participants in the control condition received an information booklet at T1 containing psychoeducation and sleep hygiene information (e.g. healthy sleep habits regarding caffeine, light, and alcohol, and information about typical infant sleep patterns) from the CBT-I condition without other components.

### Measures

Selected measures were well normed for the general population so outcomes from this study can be compared with adults in other life stages. Psychometric properties of each measure can be found in the Protocol [42].

#### Primary and Secondary Outcomes.

The primary outcome was the average scores on the ISI across T3, T4, and T5. As shown in previous data, sleep undergoes major changes during the postpartum period, and the effects of behavioral/educational sleep interventions may have different impacts on maternal sleep at different perinatal timepoints [52]. Therefore, we use ISI scores across postpartum time points to estimate cumulative, total postpartum insomnia symptom burden. The ISI [43] is a seven-item self-report measure of insomnia disorder symptom severity. Scores range from 0 to 28, with 0–7 indicating absence of insomnia, 8–14 sub-threshold insomnia, 15–21 moderate clinical insomnia, and 22–28 severe clinical insomnia [53].

Secondary outcomes included: self-reported sleep behaviors over the past week (e.g. sleep duration, onset latency, wake after sleep onset, and sleep efficiency), adapted from the Consensus Sleep Diary [54], and the PROMIS Sleep Disturbance, Sleep-Related Impairment [55], Depression and Anxiety [56]. All PROMIS measures were administered using computer adaptive tests, and generated T-scores, with a general population mean of 50 and a standard deviation of 10.

The Insomnia Module of the DSISD [45] was used to identify (1) rates of DSM-5 insomnia disorder and (2) clinically significant perinatal sleep disruption at T1 and T4; participants were also asked at T4 to recall episodes of sleep dissatisfaction between T1 and T4 and retrospectively complete the Insomnia Module for each episode. As outlined in our previous work [57], both insomnia disorder and perinatal sleep disruption met Criteria A-C and F-H of the DSM-5 insomnia disorder criteria regarding frequent and distressing sleep difficulties (excluding the 3-month duration criteria), but differed on the response to Criteria E regarding adequate sleep opportunity. That is, participants with insomnia disorder met Criteria E, with sleep complaints persisting despite adequate sleep opportunity (e.g. “I toss and turn, and it takes ages to fall asleep, even when my baby is sleeping”*).* Those with perinatal sleep disruption did not meet Criteria E, indicating that sleep difficulties did not persist when presented with adequate sleep opportunities (e.g. they did not experience poor sleep quantity/quality when they did not need to attend to the baby at night). We report both insomnia disorder and significant perinatal sleep disruption because they are both highly relevant to perinatal wellbeing [57], and are different domains targeted by the two intervention conditions.

All telephone interviews were recorded and a subset (i.e. 7% of T1 and 100% of T4) were assessed for reliability by an independent rater (MAS or NQ). No discrepancies were identified by the independent rater for the T1 telephone interviews, and discrepancies identified for the T4 telephone interviews were reconciled via discussion.

#### Other Factors.

Infant sleep duration and quality were measured with the Brief Infant Sleep Questionnaire (BISQ) [58]. The original scale has been revised (BISQ-R) [59], and in line with recommendations by Mindell and colleagues [60] this study included 5 additional items from the BISQ-R to allow for calculation of subscores regarding: (1) infant sleep, and (2) parents’ perceptions of their infant’s sleep. Total scores were generated and scaled from 0 to 100, with higher scores suggesting better sleep quality and more positive perceptions of infant sleep [60].

Beliefs and attitudes about sleep were measured using the Dysfunctional Beliefs and Attitudes about Sleep Scale (DBAS-16) [61]. Participants’ perceived credibility and expectancy of treatment was assessed via the Credibility Expectancy Questionnaire [62] with higher scores reflecting greater credibility and expectancy by the participant, and program acceptability was measured by the Client Satisfaction Questionnaire [63].

Due to the COVID-19 (Coronavirus) pandemic outbreak occurring during data collection, this study made one change to preregistered outcome measures by adding questions to capture and describe personal experiences with COVID-19 and its potential impact on project participation. All participants who completed assessments after July 2020 were asked to complete the questionnaire.

Rates of intervention delivery were collected by the research team, who documented when intervention materials or devices were delivered or not delivered. Reasons for participants not receiving the intervention were also documented (e.g. not responding to contact).

Adverse events were monitored during telephone contact at T1 and T3 by asking participants to verbally report any challenges experienced that were relevant to the interventions, and via questionnaire at T2, T4, and T5, with participants asked to answer “Yes” or “No” regarding whether they had experienced “undesirable events/effects experienced as a result of implementing the strategies discussed during the project.” If “Yes,” they were asked to provide details in a free-form text response. The research team were immediately notified if a participant selected “Yes,” and responses were discussed with the principal investigator on a case-by-case basis to decide if follow-up actions were required.

### Statistical analysis

Power analyses were carried out for comparisons between either the intervention condition with the control condition. Based on a priori power analysis assuming 10% missing data at each follow-up, randomizing 38 participants to each group was powered adequately at 80% (two-tailed *α* = 0.05) to detect a medium to large effect size (ES; *d *= 0.7). The ES estimation was based on our previous trial using the CBT-I intervention [27].

All analyses were conducted on an intention-to-treat basis and carried out using R 4.2.0 [64]. Descriptive statistics were used to characterize baseline demographic characteristics, rates of missing data over time, and outcomes over time. Baseline group differences were tested using *t*-tests or χ^2^ tests.

To examine group differences in primary and secondary outcomes, separate multiple regression analyses were conducted with treatment conditions as the independent variable, and the outcome of interest as the dependent variable, adjusting for baseline levels of the outcome [65] and stratification for each participant; linear regressions were carried out for continuous variables (e.g. ISI) and Poisson regressions were carried out for count data (e.g. the number of insomnia episodes). Following the American Statistician Association’s recommendation not to base scientific conclusions only on whether a *p*-value passes a specific threshold [66], we present ESs and 95% confidence intervals along with *p*-values for group differences at each time point. ESs between conditions at each time point were adjusted, standardized mean differences, standardized by residual variance, comparable to within-person effects. An ES of ~0.2 was considered small, ~0.5 was considered medium, and ~0.8 was considered large.

Missing data were addressed using multiple imputations through chained equations with a fully conditional specification [67, 68] using the mice package [69]. The multiple imputation model included data from 127 randomized participants, and for each variable being imputed, predictors were: group allocation, age, mental health history, the same variable from other time points, and related outcomes at the same time point. The maximum number of iterations was set at 50 to ensure good convergence. A total of 100 multiply imputed datasets were generated and results were pooled.

## Results

### Participant characteristics

**Figure 1.**
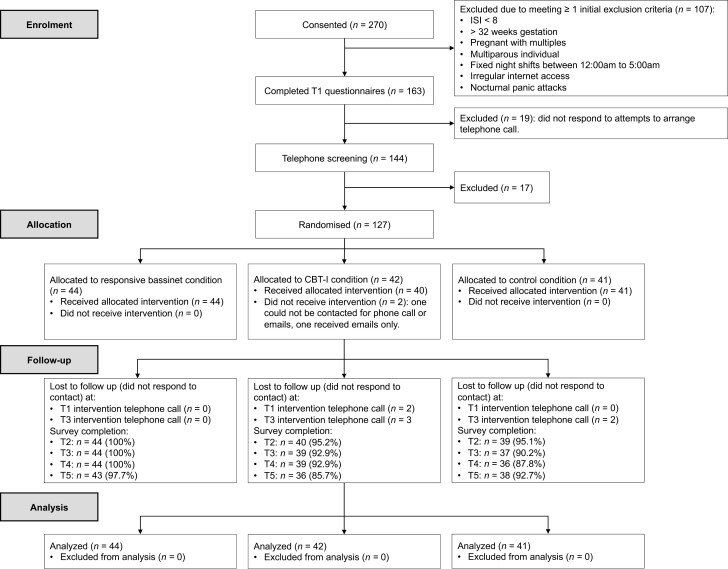
CONSORT diagram of participant flow. ISI, Insomnia Severity Index; CBT-I, Cognitive Behavioral Therapy for Insomnia; T1 = 26–32 weeks pregnancy; T2 = 35–36 weeks pregnancy; T3 = 2 months postpartum; T4 = 6 months postpartum; T5 = 12 months postpartum.

Between 30 September 2019 and 9 December 2020, 270 participants (100% female) gave informed consent and were screened for eligibility (see participant flow in Figure 1). A total of 127 participants were randomized to the RB (*n *= 44), CBT-I (*n *= 42), or control (CTRL; *n *= 41) condition, exceeding the recruitment target of 114 due to a large number of participants who signed up to the study in early pregnancy and followed through with participation. There were no stopping guidelines or interim analyses and the trial stopped due to achievement of recruitment target. Rates of missing data were low: among the participants randomized, 123 participants (96.9%) completed surveys at the pregnancy endpoint (T2), 119 participants (93.7%) completed surveys at T4 (immediately post-intervention), and 117 (92.1%) participants completed surveys at T5 (12-month postpartum follow-up). In total, 106 participants (83.5%) completed surveys for all five-time points. The analyses below include all 127 randomized participants.

Table 2 shows the sample characteristics at baseline. Participants had an average age of 32.62 years (*SD* = 3.49), and a majority of the sample had completed university education (83.5%), were white (84.3%), worked full-time (79.5%), had a household income ≥ 130 k AUD (76.3%) which is above the median household income in Victoria, Australia [70], and were married or in a de facto relationship (98.4%). Thirty (23.6%) participants reported having past or current mental health conditions. The sample reported a baseline ISI score of 13.94 (*SD *= 3.77), just under the “clinical insomnia” threshold of 15, and the mean sleep efficiency (i.e. self-reported total sleep time divided by total time in bed) was 76.1%. Based on structured clinical interviews, 80 (63.0%) and 21 (16.5%) participants met the criteria for DSM-5 insomnia disorder (excluding the 3-month duration criteria) and significant perinatal sleep disruption, respectively. The conditions were comparable on baseline characteristics, including demographics, primary and secondary outcomes, treatment credibility, and expectancy (all *p*-values ≥ .093, all ES ≤ 0.22), with differences observed for sleep onset latency (SOL; *p* = .046, ES = 0.05; higher in CTRL). At 6 months postpartum (T4), the number of hours participants worked for payment/profit ranged from 0 to 42 hours (*M *= 2.87, *SD *= 7.87), with 81.51% of participants working 0 hours per week. At 12 months postpartum (T5), the number of hours participants worked for payment/profit ranged from 0 to 43 (*M *= 17.89, *SD *= 13.88), and 29.91% of participants worked 0 hours per week.

**Table 2.** Descriptive Statistics of Baseline Sample Characteristics

	*N* = 127	Control (*n* = 41)	Responsive bassinet (*n* = 44)	CBT-I (*n* = 42)
Age in years, *M* (SD)	32.62 (3.49)	32.93 (4.07)	32.40 (3.36)	32.56 (3.05)
Gestation in weeks at baseline, *M* (SD)	27.39 (2.17)	27.05 (2.04)	27.49 (2.10)	27.63 (2.37)
Infant gestation in weeks at delivery, *M* (SD)	39.70 (1.30)	39.53 (1.38)	39.67 (1.43)	39.89 (1.08)
*Race*				
White, *n* (*%*)	107 (84.3)	34 (82.9)	37 (84.1)	36 (85.7)
Asian, *n* (*%*)	18 (14.2)	7 (17.1)	6 (13.6)	5 (11.9)
Other, *n* (*%*)	2 (1.6)	0 (0.0)	1 (2.3)	1 (2.4)
*Relationship status*				
Married/De facto, *n* (*%*)	125 (98.4)	40 (97.6)	44 (100.0)	41 (97.6)
Single, *n* (*%*)	2 (1.6)	1 (2.4)	0 (0.0)	1 (2.4)
*Employment*				
Working fulltime, *n* (*%*)	101 (79.5)	34 (82.9)	35 (79.5)	32 (76.2)
Working part-time, *n* (*%*)	21 (16.5)	5 (12.2)	9 (20.5)	7 (16.7)
Not working, *n* (*%*)	5 (3.9)	2 (4.9)	0 (0.0)	3 (7.1)
*Annual Household Income (AUD), n (%)*				
<$52k	4 (3.4)	0 (0.0)	1 (2.3)	3 (8.1)
≥$52k and <$78k	1 (0.8)	0 (0.0)	0 (0.0)	1 (2.7)
≥$78k and <$104K	7 (5.9)	2 (5.3)	3 (7.0)	2 (5.4)
≥$104k and <130k	16 (13.6)	9 (23.7)	4 (9.3)	3 (8.1)
≥$130k and <$156k	27 (22.9)	7 (18.4)	12 (27.9)	8 (21.6)
≥$156k	63 (53.4)	20 (52.6)	23 (53.5)	20 (54.1)
*Education*				
Less than tertiary education, *n* (*%*)	21 (16.5)	9 (22.0)	6 (13.6)	6 (14.3)
Bachelor, *n* (*%*)	41 (32.3)	14 (34.1)	19 (43.2)	8 (19.0)
Postgraduate, *n* (*%*)	65 (51.2)	18 (43.9)	19 (43.2)	28 (66.7)
*Mental health history*				
None, *n* (*%*)	97 (76.4)	30 (73.2)	33 (75.0)	34 (81.0)
Current or past, *n* (*%*)	30 (23.6)	11 (26.8)	11 (25.0)	8 (19.0)
Insomnia Severity Index, *M* (SD)	13.94 (3.77)	13.83 (3.51)	13.93 (3.75)	14.07 (4.09)
PROMIS sleep disturbance, *M* (SD)	58.36 (5.35)	57.54 (5.34)	58.96 (5.24)	58.53 (5.49)
PROMIS sleep-related impairment, *M* (SD)	61.01 (6.48)	60.88 (6.11)	61.54 (6.47)	60.58 (6.94)
Time-in-bed (hrs), *M* (SD)	8.92 (0.92)	8.94 (1.03)	8.99 (0.89)	8.84 (0.86)
Total sleep time (hrs), *M* (SD)	6.74 (1.11)	6.69 (1.38)	6.78 (0.91)	6.76 (1.02)
Sleep onset latency (mins), *M* (SD)	40.92 (33.37)	51.10 (39.50)	38.45 (35.55)	33.57 (20.22)
Sleep efficiency (%), *M* (SD)	76.10 (13.03)	75.09 (14.49)	76.10 (12.16)	77.07 (12.64)
Insomnia disorder ^a^, *n* (%)	80 (63.0)	26 (63.4)	26 (59.1)	28 (66.7)
Significant perinatal sleep disruption ^a^, *n* (%)	21 (16.5)	5 (12.2)	7 (15.9)	9 (21.4)
DBAS, *M* (SD)	4.59 (1.45)	4.58 (1.40)	4.68 (1.50)	4.50 (1.48)
PROMIS depression, *M* (SD)	50.52 (5.86)	51.21 (6.68)	50.42 (5.61)	49.97 (5.32)
PROMIS anxiety, *M* (SD)	54.39 (6.56)	55.68 (7.43)	54.29 (6.21)	53.25 (5.90)
CEQ: credibility	6.85 (1.22)	6.74 (1.24)	6.96 (1.35)	6.83 (1.05)
CEQ: expectancy	−0.01 (0.91)	0.02 (0.98)	0.12 (0.95)	-0.18 (0.80)

*M* (mean) and *SD* (standard deviation) are presented for continuous variables, and *n* (%) are presented for categorical variables.

^a^Established using structured clinical interview, where insomnia disorder met all DSM-5 criteria (excluding the 3-month duration criteria), and perinatal sleep disruption met the same criteria except the sleep opportunity criteria (Criteria E), indicating that sleep difficulties did not persist when presented with adequate sleep opportunity. CBT-I, Cognitive Behavioral Therapy for Insomnia; CEQ, Credibility Expectancy Questionnaire.

### Acceptability and adverse events

Both interventions were feasible and well-accepted. An average of 73.7% (range 59.5%–90.5%) of CBT-I intervention emails were opened by participants. Two participants did not respond to contact and did not receive the full CBT-I intervention (one did not receive the intervention phone call or emails, one received intervention emails only). During pregnancy, 13 participants received TIB restriction, and no participant required postpartum TIB restriction. Among participants allocated to RB, 100% of participants received the bassinet and the average number of days participants used the bassinet was 6.32 days per week (*SD* = 1.90) and 5.86 days per week (*SD* = 2.46) at 2 and 6 months postpartum, respectively. At 2 and 6 months postpartum, 95.5% and 88.6% of participants allocated to RB were using the bassinet at least 1 day per week.

Rates of intervention satisfaction obtained by the Client Satisfaction Questionnaire were high (total sample *M = *74.60, *SD *= 19.34); participants were most satisfied in the RB condition (*M* = 88.08, *SD* = 11.27), followed by the CBT-I (*M *= 77.32, *SD* = 14.74) and CTRL condition (*M* = 55.56, *SD* = 16.06), *p < *.001, ES = 0.48.

No related adverse events were reported in the CBT-I and CTRL conditions. At T4, two participants in the RB condition reported difficulty transitioning their infant from the bassinet to a crib. At T5, one participant reported that, in hindsight, the mobile application linked with the bassinet may have contributed to their anxiety regarding their infant’s sleep during the early postpartum period. No other adverse events were reported.

### Primary outcome

**Figure 2.**
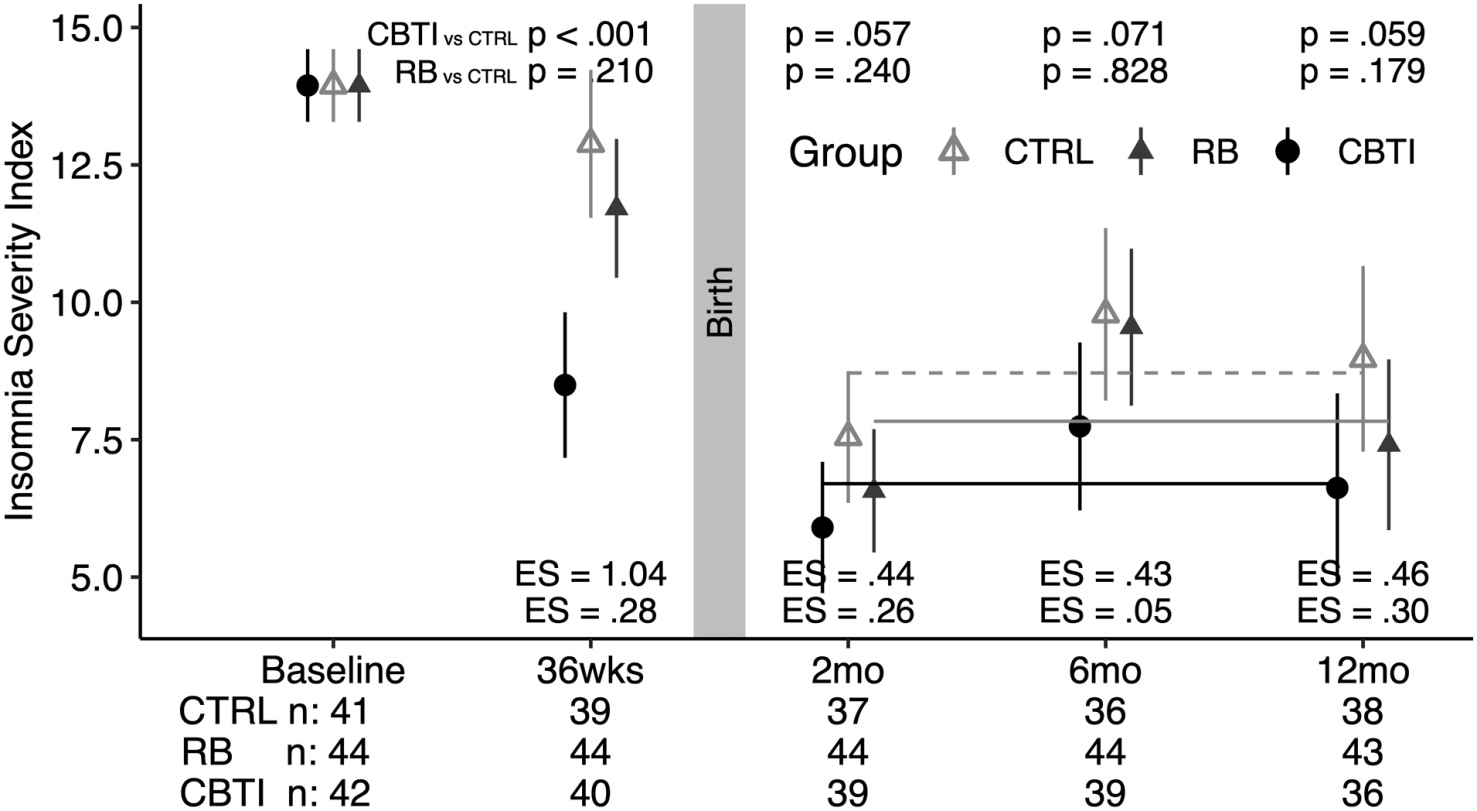
Multiple regression analyses adjusting for baseline outcomes for Insomnia Severity Index scores. Unstandardized estimates and 95% confidence intervals are shown. The primary outcome is average ISI scores across T3, T4, and T5 (represented in gray dashed line for CTRL, gray solid line for RB, and black solid line for CBT-I); CBT-I condition reported lower average ISI during the postpartum period compared to the CTRL (2.01 point ISI difference; *p = *.014, ES = 0.56); differences in the average ISI scores across T3-T5 were small between the RB and CTRL (0.87 point ISI difference; *p* = .270, ES = 0.25). See Supplementary Table S1 for numeric summary. ES, effect size; CTRL, control condition; RB, responsive bassinet condition; CBTI, Cognitive Behavioral Therapy for Insomnia condition.

Findings on the primary outcome, the ISI, are presented in the main text, with findings from multiple regression analyses visualized in Figure 2 and detailed numeric summaries presented in Supplementary Table S1.

The primary outcome is average ISI scores across T3-T5 representing postpartum insomnia symptom severity (see horizontal lines in Figure 2). Participants in the CBT-I condition reported lower insomnia symptom severity during the postpartum period compared to the CTRL with a medium ES (2.01 point ISI difference; *p = *.014, ES = 0.56). Differences in the average ISI scores across T3–T5 were small between the RB and CTRL (0.87 point ISI difference; *p* = .270, ES = 0.25).

Exploring group differences on the ISI at individual time points: at the pregnancy post-baseline time point (T2), participants in the CBT-I condition (vs. CTRL) had lower scores on the ISI with a large ES (4.38 point ISI difference; *p* < .001, ES = 1.04); differences between RB and CTRL were small (1.17 point ISI difference; *p* = .210, ES = 0.28). During postpartum time points (T3, T4, and T5) and compared to CTRL, the CBT-I condition reported lower ISI scores at all time points, with ISI differences ranging from 1.65 to 2.35, *p*-values ranging from .057 to .071 and ES ranging from .43 to .46; differences in ISI scores between the RB and CTRL conditions were small, with ISI differences ranging from 0.23 to 1.56, *p*-values ranging from .179 to .828 and ES ranging from 0.05 to 0.30.

At T4, based on structured clinical interviews (Supplementary Table S2), rates of current insomnia disorder (excluding the 3-month duration criteria) were 10.8%, 20.5%, 14.7% for the CBT-I, RB, and CTRL conditions, respectively (logistic regression: *p = *.623 for CBT-I vs CTRL, *p* = .513 for RB vs. CTRL). The proportion of participants who met criteria for perinatal sleep disruption were 28.6%, 29.5%, and 24.4% for the CBT-I, RB, and CTRL conditions, respectively (logistic regression: *p* = .783 for CBT-I vs CTRL, *p* = .990 for RB vs CTRL).

As outlined in the Methods, participants were asked to recall episodes of sleep dissatisfaction between T1 and T4 and retrospectively complete the Insomnia Module for each episode. For the period between T1 and T4, the number of insomnia disorder episodes was less for CBT-I (83.3% had 0 episodes, 16.7% had 1 episode) than CTRL (68.3% had 0, 24.4% had 1, 7.3% had 2 episodes; Poisson regression: *p* =.063), as was the average duration of insomnia disorder episodes (*M* = 0.74 weeks for CBT-I, *M* = 3.13 weeks for CTRL; *p* =.037, ES = 0.46 for CBT-I vs CTRL). RB and CTRL were comparable regarding the number (*p* =.545) and duration (*p* =.670, ES = 0.09) of insomnia disorder episodes.

### Secondary Outcomes

Secondary maternal sleep outcomes, infant sleep, and maternal symptoms of depression and anxiety were of an exploratory nature and are described in the Supplement (including Figure S1 and Table S1). Briefly, findings on maternal PROMIS sleep disturbance, sleep-related impairment, SOL, SE, and DBAS resemble those on the ISI, favouring CBT-I over CTRL. The RB and CTRL conditions were comparable in most maternal sleep outcomes, except at T4, the RB had longer TST and higher SE (both medium effects) compared to CTRL. On most infant sleep outcomes, the CBT-I and RB conditions reported minimal differences from CTRL at all time points, except small to medium differences between RB and CTRL on infant SOL, favouring RB. On maternal mood, there were small to medium differences when comparing both CBT-I and RB to CTRL at postpartum time points, favouring CBT-I and RB conditions; differences in anxiety symptoms were minimal.

### Impact of COVID-19 on participation

Of the 110 participants who completed the COVID-19 impact questionnaire at T5, 45 (40.9%) reported that the pandemic was present for their whole participation in the project, 22 (20.0%) reported that the pandemic was present for part of their participation, and 43 (39.1%) reported that none of their participation was affected by the pandemic. High proportions of participants reported that the pandemic had a somewhat to extremely negative impact on their mental health (76.4%), physical health (47.3%), loved ones’ health and wellbeing (70.9%), finances (30.9%), employment (22.7%), and relationships with others (58.2%). A small proportion of participants (10.9%) reported that the pandemic had a somewhat to extremely negative impact on their ability to use strategies/resources provided by the trial, with most participants (81.8%) indicating that the pandemic had no impact on their ability to use strategies/resources provided by the project.

## Discussion

This randomized-controlled trial investigated whether two interventions targeting different mechanisms (i.e. infant-related maternal sleep disruption as a precipitator, and unhelpful sleep-related cognitions/behaviors as perpetuators) ameliorate the development of postpartum insomnia. Both interventions were feasible and well-accepted. Compared to a control condition, CBT-I but not the RB condition prevented postpartum insomnia, with medium effects on postpartum insomnia symptoms. Exploratory analyses suggest that these two interventions may have a different impact on different aspects of sleep, and both showed small to medium effects on postpartum depressive symptoms that warrant future research.

CBT-I targeting perpetuating factors of insomnia ameliorated insomnia symptoms during both late pregnancy and across the first postpartum year among first-time birthing parents at risk of postpartum insomnia. Practically speaking, the average ISI scores across postpartum time points fell into the “absence of insomnia” category for the CBT-I condition and remained “sub-threshold insomnia” for the control condition, with moderate ESs observed. Exploratory analyses showed clinically meaningful differences at postpartum time points for (1) sleep disturbance (e.g. based on the PROMIS Sleep Disturbance, at 6 months postpartum CBT-I participants were in the 55th percentile of the general population norm for sleep disturbance compared to the 75th percentile for control), (b) sleep-related impairment (e.g. based on the PROMIS Sleep-Related Impairment at 12 months postpartum, participants who received CBT-I were in the 58th percentile of the general population norm compared to the 73rd percentile for control), and (c) dysfunctional beliefs and attitudes about sleep (e.g. at 6 months postpartum, the CBT-I condition reported a 32.0% reduction in dysfunctional sleep beliefs and attitudes compared to baseline, whereas the control condition reported reductions of 13.8%).

These findings strengthen existing literature on the efficacy of CBT-I for perinatal insomnia [27–30], and provide additional evidence for the long-term benefits of CBT-I on postpartum insomnia prevention and sleep-related wellbeing. Similar to previous work [27], insomnia diagnoses based on structured interviews did not differ between CBT-I and control at 6 months postpartum; however, across the first 6 postpartum months, early applications of CBT-I were associated with shorter and fewer episodes of insomnia disorder.

CBT-I and control reported reductions in self-reported maternal sleep duration from late pregnancy to 2 months postpartum, a trend that is often observed during the transition to parenthood [8]. Maternal sleep duration in the RB condition appeared in line with pregnancy timepoints at 2 months postpartum before increasing at 6 months postpartum. Preliminary findings showed that the RB condition reported longer self-reported maternal sleep duration (by an average of ~41 minutes per night) and higher sleep efficiency (medium ESs) than control, which requires further research. Despite these differences, the RB did not mitigate insomnia symptoms in this study. Unlike the CBT-I condition, the RB and control were comparable at all time points regarding dysfunctional beliefs and attitudes about sleep, which may have contributed to insomnia development. Whilst it is possible that the reduction in maternal sleep deprivation/disruption was not sufficient in magnitude to prevent postpartum insomnia, findings from this study support the notion that addressing precipitating factors without addressing perpetuating factors is likely insufficient to prevent insomnia in the perinatal context.

Several preliminary findings in secondary analyses warrant future research, for example, the effects of the RB on infant sleep outcomes, and the effects of both CBT-I and RB on maternal mood will require larger, adequately powered randomized controlled trials. A larger sample size will also allow statistical modeling of how different precipitating and perpetuating factors influence the development of insomnia over time.

The following sample characteristics may limit the generalizability of our findings: (1) participants were first-time birthing parents; given that parity plays an important role in maternal sleep [20], findings may not be generalized to multiparous populations and future research is needed regarding intervention efficacy in multiparous individuals; (2) the sample were mostly white, university-educated, and in stable relationships, and studies on ethnically and socioeconomically diverse samples are much needed; (3) findings may not generalize to individuals with severe psychiatric and physical health conditions, as these were excluded from the study.

This study was not powered for a non-inferiority comparison between the CBT-I and RB conditions and did not have the capacity to test the interaction of each intervention condition using a factorial design. This is a consideration for future research. Furthermore, this study was powered based on insomnia symptoms as the primary outcome; findings on secondary outcomes such as symptoms of depression and anxiety were exploratory, and were likely underpowered. Also, as specified in the prospective registration, we did not carry out alpha correction for multiple comparisons. If alpha correction were to be made, the primary outcome finding continues to hold based on *p*-value threshold, as *p*-value for the primary outcome (ISI scores across T3–T5) is 0.014 (< 0.025, which is 0.05/2) between CBT-I and control condition, and the difference between RB and control condition will remain nonsignificant.

This study utilized self-report and not objective measures of maternal and infant sleep. Whilst maternal reports of infant sleep correlate with objective measures such as actigraphy [58], findings also suggest that agreement between actigraphy and maternal reports is low regarding the number and length of infant nocturnal awakenings [71]. Furthermore, it is possible that participants not blind to their allocated conditions and nonspecific therapeutic factors (e.g. time with researcher in CBT-I condition, and receiving a bassinet in the RB condition), rather than the intervention themselves, may have contributed to the observed differences; however, the observed group differences between CBT-I and CTRL were comparable in size to that in our previous RCT [27], which the CBT-I intervention was based on, and the comparison condition received time and attention matched intervention with participants blind to which condition was “active.” Future studies using both self-report and objective measures of maternal and infant sleep are needed.

This study did not collect data on how participants used specific features of the bassinet, such as infant sleep location when the bassinet was not in use, the use of white noise, or the infant sleep report via the mobile application. Therefore, how each feature contributed to the outcomes requires future research. Further, data regarding the age of transition to a standard crib was not collected, nor was difficulty transitioning infants from a bassinet to a crib between 6 and 12 months postpartum, both of which require future research.

Although participants reported few related adverse events/effects while participating in the project, this study did not collect data after 12 months postpartum. Therefore, the long-term effects of either condition, positive or negative, were not assessed. Furthermore, the average opening rate for CBT-I intervention emails was 73.7%, somewhat lower than rates observed in a previous study (e.g. 82.3% [27]). It is possible that sample differences (individuals with insomnia symptoms in this study versus a community sample in the previous study) and the COVID-19 pandemic may have contributed to this difference.

Furthermore, this study did not measure outcomes in fathers and partners of participants. This requires further research as fathers report significant insomnia and sleepiness during the perinatal periods, and poor sleep in fathers is associated with depression and impaired family functioning [72].

This study coincided with the COVID-19 pandemic. Although only a small proportion of participants (approximately 10%) reported a reduced capacity to use strategies/resources provided by the trial, high proportions of participants reported adverse effects on their mental and physical health, relationships, and finances due to COVID-19; these may have impacted how findings could be generalized to a different societal context.

Lastly, exploring treatment mechanisms was outside the scope of the current study, and future research should seek to identify for whom and how CBT-I and RBs improved maternal sleep and mental health.

Nevertheless, this study showed that to prevent postpartum insomnia, it is critical to address perpetuating factors of insomnia, especially via early application of CBT-I; addressing precipitating factors alone is unlikely to be sufficient. Insomnia symptoms and sleep-related well-being (e.g. daytime impairment) can be ameliorated with CBT-I, whereas preliminary findings on the RB in addressing postpartum sleep deprivation and disruption require further research. Similarly, future research is needed to further examine the effects of CBT-I and RBs on postpartum depressive symptoms, especially in individuals at risk of postpartum depression.

## Supplementary Material

zsae106_suppl_Supplementary_Material

## Data Availability

De-identified data from this trial will be shared on reasonable request to the corresponding authors.
