# Supplementary material for: Preventing postpartum insomnia: findings from a three-arm randomized-controlled trial of cognitive behavioral therapy for insomnia, a responsive bassinet, and sleep hygiene
Source: Sleep. 2024 May 13;47(8):zsae106. doi: 10.1093/sleep/zsae106 (PMC11321850; doi:10.1093/sleep/zsae106)
Supplement: zsae106_suppl_Supplementary_Material [file zsae106_suppl_supplementary_material.docx]

Preventing postpartum insomnia: findings from a three-arm randomised controlled trial of Cognitive Behavioural Therapy for Insomnia, a responsive bassinet, and sleep hygiene.

Nina Quin^1,2^, Liat Tikotzky^3^, Laura Astbury^1^, Marie-Antoinette Spina^1^, Jane Fisher^5^, Lesley Stafford^2,4^, Joshua F. Wiley^1^, Bei Bei^1,2^.

1. Turner Institute for Brain and Mental Health, School of Psychological Sciences, Faculty of Medicine, Nursing and Health Sciences, Monash University, Victoria, Australia;
2. Women’s Mental Health Service, Royal Women's Hospital, Victoria, Australia;
3. Department of Psychology, Ben-Gurion University of the Negev, Be'er Sheva, Israel;
4. Melbourne School of Psychological Sciences, University of Melbourne, Victoria, Australia;
5. Global and Women’s Health, School of Public Health and Preventive Medicine, Monash University, Victoria, Australia.

Correspondence to: Bei Bei, DPsych(Clinical), PhD, Turner Institute for Brain and Mental Health, School of Psychological Sciences, Faculty of Medicine, Nursing and Health Sciences, Monash University, 18 Innovation Walk, Clayton Campus, Victoria 3800, Australia. [bei.bei@monash.edu](mailto:bei.bei@monash.edu).

## Exploratory Findings on Secondary Outcomes

This trial was powered for the average postpartum insomnia symptoms as the primary outcome, therefore secondary outcomes presented below are of an exploratory nature to support the interpretation of findings. Future studies are needed to further examine effect sizes described in this study.

### Maternal Sleep Outcomes

At pregnancy post-baseline time point (T2), the CBT-I condition had lower PROMIS sleep disturbance (*p* < .001, ES = 0.87), sleep-related impairment (*p* < .001, ES = 0.98), SOL (*p* = .039, ES = 0.48), DBAS (*p* < .001, ES = 1.32), and higher sleep efficiency (*p =* .004, ES = 0.67) compared to CTRL. The CBT-I and CTRL conditions were comparable on depression and anxiety symptoms (*p*-value ranges .530 to .857, ES ranges 0.04 to 0.14). The RB and CTRL conditions were comparable on all maternal sleep measures at T2 (*p*-value ranges .365 to .971, ES ranges 0.01 to 0.20).

At all three postpartum time points, the CBT-I condition reported lower sleep disturbance, sleep-related impairment, and DBAS, and higher sleep efficiency (except at T3) compared to CTRL (*p*-value ranges <.001 to .564, ES ranges 0.13 to 0.86). Differences between CBT-I and CTRL for maternal SOL and TST at postpartum time points were small (*p*-value ranges .221 to .773, ES ranges 0.07 to 0.29), except medium effects were observed for TST at T5 (*p =* .088, ES = 0.41).

The RB condition reported longer TST (approximately 41 min longer on average; *p* = .008, ES = 0.62) and higher sleep efficiency (*p* = .015, ES = 0.55) compared to CTRL at T4, but differences at T3 and T5 were small for both TST (*p*-value ranges .309 to .907, ES ranges 0.03 to 0.23) and sleep efficiency (*p*-value ranges .381 to .719, ES ranges 0.08 to 0.20). The RB and CTRL were comparable for DBAS at postpartum time points (*p*-value ranges .545 to .740, ES ranges 0.08 to 0.14) and minimal to small effects were observed between RB and CTRL for maternal SOL, sleep disturbance, and sleep-related impairment at postpartum time points (*p*-value ranges .103 to .981, ES ranges 0.01 to 0.37).

### Infant Sleep

Regarding infant sleep outcomes (see Table S1), the CBT-I and CTRL conditions reported minimal differences on infant sleep variables at all time points (*p*-value ranges .117 to .980, ES ranges 0.01 to 0.39), and variables that exhibited small effects (e.g., infant TST during the day) favoured the CTRL condition, with the exception of infant SOL. Compared to the CTRL, the RB condition reported lower infant SOL at all time points, with small to medium effects at T3 (*p* = .051, ES = 0.45), T4 (*p* = .092, ES = 0.39), and T5 (*p* = .040, ES = 0.49). Minimal to small effects were observed between the RB and CTRL condition at T3-T5 for the number and duration of infant night awakenings, longest episode of nighttime sleep, infant TST during the night, day, and over 24 hours, as well as Infant Sleep and Parent Perception subscales of the BISQ-R (*p*-value ranges .146 to .926, ES ranges 0.02 to 0.33).

### Maternal Depression and Anxiety Symptoms

Participants in the CBT-I condition reported lower depressive symptoms than CTRL at all postpartum time points, with a small effect size at T3 (*p* = .174, ES = 0.32), and medium effects at T4 (*p* = .047, ES = 0.47) and T5 (*p* = .084, ES = 0.42). Similarly, the RB condition reported lower postpartum depressive symptoms than CTRL, with small effects at T3 (*p* = .179, ES = 0.31) and T5 (*p* *=* .176, ES = 0.32), and a medium effect at T4 (*p* = .077, ES = 0.40). Anxiety symptoms were comparable between CBT-I and CTRL at T3 (*p* = .542, ES = 0.14) and small effects were observed at T4 and T5 (*p*-value ranges .171 to .338, ES ranges 0.24 to 0.33). RB and CTRL were comparable for anxiety symptoms at all postpartum time points (*p*-value ranges .456 to .660, ES ranges 0.10 to 0.17).

**Figure S1**. Multiple regression analyses adjusting for baseline outcomes. Unstandardized estimates and 95% confidence intervals are shown. See Supplementary Table S1 for numeric summary. ES, effect size; CTRL, control condition; RB, responsive bassinet condition; CBTI, Cognitive Behavioural Therapy for Insomnia condition.

**Table S1**

*Post-Baseline Adjusted Means [95% Confidence Interval] and Findings from Multiple Regression Analyses.*

|  | **Control (CTRL)**  (*n* = 41) | **Responsive Bassinet (RB)**  (*n* = 44) | **CBT-I**  (*n* = 42) | **CBT-I vs. CTRL**  *p* value, effect size | **RB**  **vs. CTRL**  *p* value, effect size |
| --- | --- | --- | --- | --- | --- |
| **Insomnia Severity Index** | | | | | |
| T2 | 12.88 [11.54, 14.23] | 11.71 [10.45, 12.97] | 8.50 [7.17, 9.82] | < .001, -1.04 | .210, -0.28 |
| T3 | 7.55 [6.35, 8.75] | 6.57 [5.45, 7.69] | 5.90 [4.71, 7.10] | .057, -0.44 | .240, -0.26 |
| T4 | 9.78 [8.21, 11.35] | 9.55 [8.12, 10.98] | 7.74 [6.21, 9.27] | .071, -0.43 | .828, -0.05 |
| T5 | 8.97 [7.28, 10.66] | 7.41 [5.85, 8.96] | 6.62 [4.90, 8.34] | .059, -0.46 | .179, -0.30 |
| T3-T5 | 8.71 [7.57, 9.86] | 7.84 [6.77, 8.91] | 6.70 [5.59, 7.81] | .014, -0.56 | .270, -0.25 |
| **PROMIS Sleep Disturbance** | | | | | |
| T2 | 57.79 [55.93, 59.65] | 56.61 [54.85, 58.36] | 52.66 [50.83, 54.50] | < .001, -0.87 | .365, -0.20 |
| T3 | 52.85 [50.68, 55.02] | 50.98 [48.96, 53.00] | 48.18 [46.04, 50.32] | .003, -0.69 | .215, -0.28 |
| T4 | 56.94 [54.56, 59.32] | 55.70 [53.53, 57.87] | 51.31 [48.99, 53.64] | .001, -0.78 | .448, -0.17 |
| T5 | 53.84 [51.51, 56.16] | 51.60 [49.48, 53.72] | 49.90 [47.55, 52.24] | .019, -0.56 | .163, -0.32 |
| **PROMIS Sleep Related Impairment** | | | | | |
| T2 | 58.59 [56.62, 60.56] | 58.54 [56.68, 60.40] | 52.46 [50.51, 54.42] | < .001, -0.98 | .971, -0.01 |
| T3 | 57.02 [55.09, 58.95] | 55.40 [53.63, 57.18] | 53.76 [51.84, 55.69] | .019, -0.55 | .224, -0.27 |
| T4 | 58.00 [55.49, 60.50] | 56.55 [54.23, 58.87] | 54.13 [51.68, 56.58] | .032, -0.50 | .403, -0.19 |
| T5 | 56.26 [53.77, 58.76] | 53.44 [51.12, 55.76] | 52.07 [49.50, 54.64] | .023, -0.55 | .103, -0.37 |
| **Total Sleep Time (hours)** | | | | | |
| T2 | 6.74 [6.39, 7.09] | 6.71 [6.38, 7.03] | 7.03 [6.68, 7.37] | .250, 0.26 | .896, -0.03 |
| T3 | 6.43 [6.04, 6.82] | 6.70 [6.35, 7.05] | 6.35 [5.97, 6.73] | .773, -0.07 | .309, 0.23 |
| T4 | 6.30 [5.93, 6.67] | 6.98 [6.65, 7.31] | 6.60 [6.25, 6.96] | .246, 0.27 | .008, 0.62 |
| T5 | 6.82 [6.48, 7.15] | 6.79 [6.48, 7.10] | 7.23 [6.89, 7.57] | .088, 0.41 | .907, -0.03 |
| **Sleep Onset Latency (min)** | | | | | |
| T2 | 39.19 [31.29, 47.08] | 36.23 [28.82, 43.63] | 27.30 [19.47, 35.14] | .039, -0.48 | .590, -0.12 |
| T3 | 20.16 [15.56, 24.76] | 19.06 [14.86, 23.25] | 16.81 [12.25, 21.37] | .310, -0.24 | .727, -0.08 |
| T4 | 26.60 [20.18, 33.02] | 26.70 [21.02, 32.39] | 21.07 [14.96, 27.18] | .221, -0.29 | .981, 0.01 |
| T5 | 26.16 [20.20, 32.12] | 19.58 [14.02, 25.14] | 24.07 [18.02, 30.12] | .631, -0.11 | .113, -0.36 |
| **Sleep Efficiency (%)** | | | | | |
| T2 | 71.88 [68.28, 75.47] | 73.39 [70.03, 76.75] | 79.45 [75.91, 82.98] | .004, 0.67 | .544, 0.13 |
| T3 | 69.07 [64.36, 73.78] | 70.24 [65.88, 74.60] | 67.14 [62.48, 71.80] | .564, -0.13 | .719, 0.08 |
| T4 | 68.69 [64.51, 72.87] | 75.73 [71.91, 79.54] | 74.51 [70.44, 78.57] | .051, 0.46 | .015, 0.55 |
| T5 | 81.19 [77.60, 84.77] | 79.01 [75.66, 82.36] | 83.92 [80.22, 87.62] | .293, 0.25 | .381, -0.20 |
| **Dysfunctional Beliefs and Attitudes about Sleep** | | | | | |
| T2 | 4.80 [4.40, 5.19] | 4.57 [4.21, 4.93] | 3.21 [2.83, 3.58] | < .001, -1.32 | .407, -0.19 |
| T3 | 3.87 [3.49, 4.25] | 4.02 [3.69, 4.36] | 2.92 [2.55, 3.28] | < .001, -0.86 | .545, 0.14 |
| T4 | 3.95 [3.50, 4.39] | 3.83 [3.43, 4.22] | 3.06 [2.64, 3.49] | .006, -0.67 | .696, -0.09 |
| T5 | 3.77 [3.25, 4.29] | 3.66 [3.20, 4.11] | 3.05 [2.54, 3.57] | .058, -0.49 | .740, -0.08 |
| **PROMIS Depression** | | | | | |
| T2 | 47.71 [46.03, 49.39] | 49.10 [47.56, 50.64] | 46.96 [45.32, 48.60] | .530, -0.14 | .230, 0.27 |
| T3 | 49.76 [48.00, 51.52] | 48.15 [46.58, 49.72] | 48.09 [46.42, 49.76] | .174, -0.32 | .179, -0.31 |
| T4 | 51.77 [49.63, 53.91] | 49.18 [47.27, 51.10] | 48.76 [46.71, 50.81] | .047, -0.47 | .077, -0.40 |
| T5 | 51.08 [49.10, 53.07] | 49.26 [47.50, 51.01] | 48.64 [46.70, 50.58] | .084, -0.42 | .176, -0.32 |
| **PROMIS Anxiety** | | | | | |
| T2 | 52.54 [50.74, 54.33] | 53.20 [51.56, 54.84] | 52.77 [51.02, 54.51] | .857, 0.04 | .591, 0.12 |
| T3 | 49.76 [47.90, 51.62] | 50.42 [48.76, 52.07] | 50.56 [48.74, 52.38] | .542, 0.14 | .601, 0.12 |
| T4 | 53.20 [50.66, 55.74] | 51.92 [49.65, 54.18] | 50.74 [48.29, 53.18] | .171, -0.33 | .456, -0.17 |
| T5 | 52.46 [50.25, 54.68] | 51.80 [49.83, 53.77] | 50.91 [48.66, 53.16] | .338, -0.24 | .660, -0.10 |
| **Infant Sleep Onset Latency (min)** | | | | | |
| T3 | 60.80 [48.05, 73.56] | 43.86 [32.56, 55.17] | 55.02 [42.75, 67.29] | .517, -0.15 | .051, -0.45 |
| T4 | 38.97 [30.03, 47.90] | 28.73 [20.81, 36.64] | 33.95 [25.53, 42.37] | .414, -0.19 | .092, -0.39 |
| T5 | 34.23 [26.00, 42.45] | 22.95 [15.89, 30.01] | 25.38 [16.99, 33.77] | .139, -0.39 | .040, -0.49 |
| **Infant Night Awakening – Number** | | | | | |
| T3 | 2.41 [1.79, 3.02] | 2.05 [1.51, 2.58] | 2.48 [1.92, 3.04] | .856, 0.05 | .382, -0.20 |
| T4 | 2.64 [2.03, 3.25] | 3.01 [2.47, 3.55] | 2.78 [2.20, 3.36] | .736, 0.08 | .367, 0.21 |
| T5 | 1.45 [0.96, 1.94] | 1.73 [1.28, 2.17] | 1.51 [1.01, 2.00] | .878, 0.04 | .415, 0.19 |
| **Infant Night Awakening - Duration (min)** | | | | | |
| T3 | 107.26 [86.64, 127.88] | 103.98 [85.19, 122.76] | 118.73 [98.70, 138.75] | .431, 0.18 | .816, -0.05 |
| T4 | 67.88 [52.06, 83.69] | 75.68 [61.58, 89.79] | 76.27 [61.30, 91.25] | .441, 0.18 | .467, 0.17 |
| T5 | 36.64 [23.82, 49.47] | 34.80 [23.54, 46.06] | 33.36 [20.46, 46.26] | .725, -0.09 | .831, -0.05 |
| **Infant Longest Nighttime Sleep (hours)** | | | | | |
| T3 | 5.66 [5.00, 6.32] | 5.55 [4.98, 6.13] | 5.05 [4.39, 5.72] | .196, -0.32 | .800, -0.06 |
| T4 | 6.36 [5.55, 7.18] | 5.55 [4.81, 6.29] | 5.88 [5.07, 6.70] | .414, -0.19 | .146, -0.33 |
| T5 | 8.00 [6.99, 9.00] | 7.59 [6.69, 8.48] | 8.14 [7.17, 9.12] | .836, 0.05 | .547, -0.14 |
| **Infant Total Sleep Time – Night (hours)** | | | | | |
| T3 | 8.63 [8.20, 9.07] | 8.94 [8.55, 9.34] | 8.46 [8.04, 8.88] | .569, -0.13 | .300, 0.23 |
| T4 | 10.05 [9.65, 10.45] | 10.10 [9.75, 10.46] | 9.96 [9.59, 10.34] | .756, -0.07 | .840, 0.04 |
| T5 | 10.60 [10.29, 10.91] | 10.50 [10.21, 10.79] | 10.54 [10.23, 10.85] | .798, -0.06 | .655, -0.10 |
| **Infant Total Sleep Time – Day (hours)** | | | | | |
| T3 | 5.36 [4.78, 5.93] | 4.90 [4.40, 5.41] | 4.86 [4.32, 5.41] | .216, -0.29 | .245, -0.26 |
| T4 | 3.14 [2.84, 3.44] | 3.16 [2.90, 3.43] | 2.81 [2.52, 3.10] | .117, -0.37 | .909, 0.03 |
| T5 | 2.54 [2.24, 2.84] | 2.40 [2.11, 2.69] | 2.36 [2.06, 2.66] | .410, -0.19 | .514, -0.14 |
| **Infant Total Sleep Time – 24 hr period (hours)** | | | | | |
| T3 | 13.92 [13.24, 14.60] | 13.85 [13.23, 14.46] | 13.30 [12.64, 13.96] | .197, -0.30 | .873, -0.04 |
| T4 | 13.17 [12.65, 13.69] | 13.27 [12.81, 13.73] | 12.75 [12.27, 13.24] | .244, -0.27 | .781, 0.06 |
| T5 | 13.14 [12.70, 13.58] | 12.90 [12.49, 13.32] | 12.90 [12.47, 13.34] | .457, -0.17 | .446, -0.17 |
| **BISQ-R Infant Sleep Subscale** | | | | | |
| T3 | 63.46 [58.14, 68.78] | 64.40 [59.54, 69.26] | 57.67 [52.44, 62.91] | .128, -0.36 | .797, 0.06 |
| T4 | 58.76 [52.31, 65.21] | 55.50 [49.55, 61.45] | 55.78 [49.49, 62.07] | .513, -0.15 | .463, -0.16 |
| T5 | 70.64 [63.82, 77.45] | 70.20 [64.04, 76.37] | 72.49 [65.72, 79.26] | .705, 0.09 | .926, -0.02 |
| **BISQ-R Parent Perception Subscale** | | | | | |
| T3 | 69.35 [62.12, 76.58] | 72.33 [65.75, 78.91] | 69.22 [62.20, 76.24] | .980, -0.01 | .547, 0.14 |
| T4 | 60.84 [53.28, 68.39] | 61.70 [54.90, 68.51] | 63.14 [55.86, 70.41] | .664, 0.10 | .866, 0.04 |
| T5 | 73.76 [65.59, 81.93] | 71.97 [64.77, 79.18] | 78.66 [70.59, 86.73] | .397, 0.21 | .745, -0.08 |

*Note.* Post-baseline outcomes are adjusted for baseline levels; See Table 2 in the main text for values at baseline; T2 = 35-36 weeks of gestation, T3 = 2 months postpartum, T4 = 6 months postpartum, and T5 = 12 months postpartum; T3-T5 average Insomnia Severity Index score is the primary outcome of the trial. CBT-I = Cognitive Behavioural Therapy for Insomnia.

**Table S2**

*Post-Baseline Rates of Insomnia Disorder and Perinatal Sleep Disruption based on Structured Clinical Interview.*

|  | **Control (CTRL)** | **Responsive Bassinet (RB)** | **CBT-I** | **CBT-I vs. CTRL**  *p* value | **RB**  **vs. CTRL**  *p* value |
| --- | --- | --- | --- | --- | --- |
| **T4 (6 months postpartum)** | | | | | |
| Insomnia Disorder, *n* (*%*) | 5 (14.7%) | 9 (20.5%) | 4 (10.8%) | .623 | .513 |
| Perinatal Sleep Disruption, *n* (*%*) | 10 (24.4%) | 13 (29.5%) | 12 (28.6%) | .783 | .990 |
| **T1 to T4 Insomnia Disorder episodes** | | | | | |
| % with 0, 1, and 2 episodes | 68.3%, 24.4%, 7.3% | 56.8%, 38.6%, 4.5% | 83.3%, 16.7%, 0.0% | .063 | .545 |
| Episode duration, *M* [95% CI] | 3.13 [1.54, 4.73] | 3.61 [2.07, 5.16] | 0.74 [-0.84, 2.32] | .037, ES = -0.46 | .670, ES = 0.09 |

*Note.* Diagnostic status was established using structured clinical interview, with Insomnia Disorder meeting DSM-5 criteria except the 3-month duration criteria. See Methods for definition of Perinatal Sleep Disruption. Interviews conducted at T4 (6 months postpartum) regarding current complaints are presented as Number (%) and between-group differences were assessed using logistic regression. For T1-T4 Insomnia Disorder episodes, regression analyses (Poisson for number of episodes and linear regression for duration) were conducted to determine group differences. CBT-I = Cognitive Behavioural Therapy for Insomnia.

**Table S3.**

*CONSORT 2010, CONSORT-SPI 2018 and CONSORT PRO checklists.*

| **Section** | **Item #** | **CONSORT 2010 Checklist item** | **CONSORT-SPI 2018^a^ Checklist item** | **CONSORT PRO^b^ Checklist item** | **Page No.** |
| --- | --- | --- | --- | --- | --- |
| **Title and abstract** | | | | | |
|  | 1a | Identification as a randomised trial in the title |  |  | 1 |
|  | 1b | Structured summary of trial design, methods, results, and conclusions | Refer to CONSORT extension for social and psychological intervention trial abstracts | The PRO should be identified in the abstract as a primary or secondary outcome | 2 |
| **Introduction** | | | | | |
| Background and  Objectives | 2a | Scientific background and explanation of rationale |  | Including background and rationale for PRO assessment | 5-8 |
|  | 2b | Specific objectives or hypotheses | If pre-specified, how the intervention was hypothesised to work | The PRO hypothesis should be stated and relevant domains identified, if applicable | 8 |
| **Methods** | | | | | |
| Trial Design | 3a | Description of trial design (such as parallel, factorial) including allocation ratio |  |  | 8, 9 |
|  | 3b | Important changes to methods after trial commencement (such as eligibility criteria), with reasons |  |  | 15 |
| Participants | 4a | Eligibility criteria for participants | When applicable, eligibility criteria for settings and those delivering the interventions | Not PRO-specific, unless the PROs were used in eligibility or stratification criteria | 8, 9 |
|  | 4b | Settings and locations where the data were collected |  |  | 9, 10 |
| Interventions | 5 | The interventions for each group with sufficient details to allow replication, including how and when they were actually administered |  |  | 10-13 |
|  | 5a |  | Extent to which interventions were actually delivered by providers and taken up by participants as planned |  | 18, 19, Figure 1 |
|  | 5b |  | Where other informational materials about delivering the intervention can be accessed |  | 10-13 |
|  | 5c |  | When applicable, how intervention providers were assigned to each group |  | 13 |
| Outcomes | 6a | Completely defined pre-specified primary and secondary outcome measures, including how and when they were assessed |  | Evidence of PRO instrument validity and reliability should be provided or cited if available including the person completing the PRO and methods of data collection (paper, telephone, electronic, other) | 10, 14-16 |
|  | 6b | Any changes to trial outcomes after the trial commenced, with reasons |  |  | 15 |
| Sample Size | 7a | How sample size was determined |  | Not required for PRO unless it is a primary study outcome | 16 |
|  | 7b | When applicable, explanation of any interim analyses and stopping guidelines |  |  | 17 |
| **Randomisation** | | | | | |
| Sequence  generation | 8a | Method used to generate the random allocation sequence |  |  | 9 |
|  | 8b | Type of randomisation; details of any restriction (such as blocking and block size) |  |  | 9 |
| Allocation concealment mechanism | 9 | Mechanism used to implement the random allocation sequence (such as sequentially numbered containers), describing any steps taken to conceal the sequence until interventions were assigned |  |  | 9 |
| Implementation | 10 | Who generated the random allocation sequence, who enrolled participants, and who assigned participants to interventions |  |  | 9 |
| Awareness of assignment | 11a | If done, who was blinded after assignment to interventions (for example, participants, care providers, those assessing outcomes) and how |  |  | 9, 10 |
|  | 11b | If relevant, description of the similarity of interventions |  |  | 13 |
| Analytical  methods | 12a | Statistical methods used to compare groups for primary and secondary outcomes | How missing data were handled, with details of any imputation method | Statistical approaches for dealing with missing data are explicitly stated | 16, 17 |
|  | 12b | Methods for additional analyses, such as subgroup analyses and adjusted analyses |  |  | 16, 17 |
| **Results** | | | | | |
| Participant flow | 13a | For each group, the numbers of participants who were randomly assigned, received intended treatment, and were analysed for the primary outcome | Where possible, the number approached, screened, and eligible prior to random assignment, with reasons for non-enrolment | The number of PRO outcome data at baseline and at subsequent time points should be made transparent | 17, 18, Figure 1 |
|  | 13b | For each group, losses and exclusions after randomisation, together with reasons |  |  | 17, 18, Figure 1 |
| Recruitment | 14a | Dates defining the periods of recruitment and follow-up |  |  | 17 |
|  | 14b | Why the trial ended or was stopped |  |  | 17 |
| Baseline data | 15 | A table showing baseline demographic and clinical characteristics for each group | Include socioeconomic variables where applicable | Including baseline PRO data when collected | Table 2 |
| Numbers analysed | 16 | For each group, number of participants (denominator) included in each analysis and whether the analysis was by original assigned groups |  | Required for PRO results | 17, 18, Figure 1, Figure 2, Figure S1, Table S2 |
| Outcomes and estimation | 17a | For each primary and secondary outcome, results for each group, and the estimated effect size and its precision (such as 95% confidence interval) | Indicate availability of trial data | For multidimensional PRO results from each domain and time point | 18-21, Table S1, Table S2, Figure 2, Figure S1 |
|  | 17b | For binary outcomes, presentation of both absolute and relative effect sizes is recommended |  |  | No binary outcomes analysed. |
| Ancillary analyses | 18 | Results of any other analyses performed, including subgroup analyses and adjusted analyses, distinguishing pre-specified from exploratory |  | Including PRO analyses, where relevant | 20, 21, Supplement p 2-3, Figure S1, Table S2 |
| Harms | 19 | All important harms or unintended effects in each group |  |  | 18, 19 (see CONSORT harms Extension: Table S4 below) |
| **Discussion** | | | | | |
| Limitations | 20 | Trial limitations, addressing sources of potential bias, imprecision, and, if relevant, multiplicity of analyses |  | PRO–specific limitations and implications for generalisability and clinical practice | 24-26 |
| Generalisability | 21 | Generalisability (external validity, applicability) of the trial findings |  |  | 24 |
| Interpretation | 22 | Interpretation consistent with results, balancing benefits and harms, and considering other relevant evidence |  | PRO data should be interpreted in relation to clinical outcomes including survival data, where relevant | 22-26 |
|  | | | | | |
| Registration | 23 | Registration number and name of trial registry |  |  | 8 |
| Protocol | 24 | Where the full trial protocol can be accessed, if available |  |  | 8 |
| Declaration of Interests | 25a | Sources of funding and other support (such as supply of drugs), role of funders |  |  | 27 |
|  | 25b |  | Declaration of any other potential interests |  | 27 |
| Stakeholder investments | 26a |  | Any involvement of the intervention developer in the design, conduct, analysis, or reporting of the trial |  | 27 |
|  | 26b |  | Other stakeholder involvement in trial design, conduct, or analyses |  | 10 |
|  | 26c |  | Incentives offered as part of the trial |  | 10 |

This table contains items from the CONSORT 2010 checklist (with some modifications for social and psychological intervention trials) and additional items in the CONSORT-SPI 2018 extension. Empty rows in the ‘CONSORT-SPI 2018’ and ‘CONSORT PRO’ columns indicate that there is no extension to the CONSORT 2010 item.^a^ From: Montgomery, P., Grant, S., Mayo-Wilson, E., Macdonald, G., Michie, S., Hopewell, S., … CONSORT-SPI Group. (2018). Reporting randomised trials of social and psychological interventions: the CONSORT-SPI 2018 Extension. *Trials, 19*(1), 407. doi:10.1186/s13063-018-2733-1 ^b^ From: Calvert, M., Blazeby, J., Altman, D. G., Revicki, D. A., Moher, D., … CONSORT PRO Group (2013). Reporting of patient-reported outcomes in randomized trials: the CONSORT PRO extension. *JAMA, 309*(8), 814–822. doi:10.1001/jama.2013.879

**Table S4.**

*CONSORT harms extension.*

| **Section** | **Item #** | **Relevant CONSORT 2010 Checklist item** | **CONSORT Harms Extension** | **Page No.** |
| --- | --- | --- | --- | --- |
| **Title and abstract** | | | | |
|  | 1a | Identification as a randomised trial in the title | If the study collected data on harms and benefits, the title or abstract should so state | 1 |
|  | 1b | Structured summary of trial design, methods, results, and conclusions |  |  |
| **Introduction** | | | | |
| Background and  Objectives | 2a | Scientific background and explanation of rationale | If the trial addresses both harms and benefits, the introduction should so state. | 5-8 |
| Outcomes | 6a | Completely defined pre-specified primary and secondary outcome measures, including how and when they were assessed | List addressed adverse events with definitions for each (with attention, when relevant, to grading, expected vs. unexpected events, reference to standardised and validated definitions, and description of new definitions).  Clarify how harms-related information was collected (mode of data collection, timing, attribution methods, intensity of ascertainment, and harms-related monitoring and stopping rules, if pertinent) | 16 |
|  | 6b | Any changes to trial outcomes after the trial commenced, with reasons |  |  |
| **Randomisation** | | | | |
| Analytical  methods | 12a | Statistical methods used to compare groups for primary and secondary outcomes | Describe plans for presenting and analysing information on harms (including coding, handling of recurrent events, specification of timing issues, handling of continuous measures and any statistical analyses) | 16 |
|  | 12b | Methods for additional analyses, such as subgroup analyses and adjusted analyses |  |  |
| **Results** | | | | |
| Participant flow | 13a | For each group, the numbers of participants who were randomly assigned, received intended treatment, and were analysed for the primary outcome | Describe for each arm the participant withdrawals that are due to harms and the experience with the allocated treatment | 18, 19 |
|  | 13b | For each group, losses and exclusions after randomisation, together with reasons |  |  |
| Numbers analysed | 16 | For each group, number of participants (denominator) included in each analysis and whether the analysis was by original assigned groups | Provide the denominators for analyses on harms | 18, 19 |
| Outcomes and estimation | 17a | For each primary and secondary outcome, results for each group, and the estimated effect size and its precision (such as 95% confidence interval) | Present the absolute risk of each adverse event (specifying type, grade, and seriousness per arm), and present appropriate metrics for recurrent events, continuous variables and scale variables, whenever pertinent.  Describe any subgroup analyses and exploratory analyses for harms | 18, 19 |
|  | 17b | For binary outcomes, presentation of both absolute and relative effect sizes is recommended |  |  |
| Ancillary analyses | 18 | Results of any other analyses performed, including subgroup analyses and adjusted analyses, distinguishing pre-specified from exploratory |  |  |
| Harms | 19 | All important harms or unintended effects in each group |  |  |
| **Discussion** | | | | |
| Limitations | 20 | Trial limitations, addressing sources of potential bias, imprecision, and, if relevant, multiplicity of analyses | Provide a balanced discussion of benefits and harms with emphasis on study limitations, generalisability and other sources of information on harms | 22-26 |
| Generalisability | 21 | Generalisability (external validity, applicability) of the trial findings |  |  |
| Interpretation | 22 | Interpretation consistent with results, balancing benefits and harms, and considering other relevant evidence |  |  |

CONSORT 2010 checklist items have been condensed for readability: only relevant items that corresponded to CONSORT Harms items are displayed. See: Ioannidis, J. P., Evans, S. J., Gøtzsche, P. C., O'Neill, R. T., Altman, D. G., Schulz, K., … CONSORT Group. (2004). Better reporting of harms in randomized trials: an extension of the CONSORT statement. *Annals of Internal Medicine, 141*(10), 781–788. doi:10.7326/0003-4819-141-10-200411160-0000
